# Gender-specific differences in feasibility of pre-lacrimal window approach

**DOI:** 10.1038/s41598-021-87447-w

**Published:** 2021-04-08

**Authors:** A. Andrianakis, U. Moser, A. Wolf, P. Kiss, C. Holzmeister, D. Andrianakis, P. V. Tomazic

**Affiliations:** 1 Department of Otorhinolaryngology, Medical University of Graz, Graz, Austria; 2 Institute of Mathematics and Scientific Computing, University of Graz, Graz, Austria

**Keywords:** Risk factors, Signs and symptoms, Respiratory signs and symptoms, Respiratory tract diseases

## Abstract

The feasibility and surgical effort of a pre-lacrimal window approach (PLWA) depends on the width of the bony window anterior to the nasolacrimal duct. This study aimed to investigate gender-specific differences in feasibility of PLWA. A consecutive series of paranasal computed tomography scans from 50 females (n = 100) and 50 males (n = 100) were retrospectively analyzed. The primary outcome measure was the antero-posterior length of the bony pre-lacrimal window (BPLWA). The secondary outcome measure was the distribution of Simmen’s PLWA feasibility types (major, moderate and minor surgical effort). On average, males had a 1.5 mm (95% CI 0.8–2.2) significantly higher BPLW length in comparison to females [t(198) = 4.4, *p* < 0.0001]. The requirement of major surgical effort occurred 29% more frequently in females [χ^2^(1) = 17.7, *p* < 0.0001], whereas the necessity of moderate surgical effort was 21% more prevalent in males [χ^2^(1) = 8.8, *p* = 0.003]. The need of only minor surgical effort was twice as high in males compared to females [χ^2^(1) = 3, *p* = 0.081]. Our data indicates that females require more significant surgical effort during a PLWA to gain access to the maxillary sinus. These results are highly informative as a high amount of bone removal and nasolacrimal duct dislocation are associated with a higher likelihood of complications.

## Introduction

The complexity of surgical access to lesions within the maxillary sinus depends in particular on the lesion’s location^1^. Performing a type III sinusotomy provides a readily exposure of pathologies along the medial and posterior wall of the maxillary sinus^2^. Pathologies located at the anterior wall and alveolar recess of the maxillary sinus are much more challenging to access. Various surgical approaches to these difficultly accessible regions have been described. Open surgical methods such as lateral rhinotomy, Caldwell-Luc procedure or midfacial removal allow a broad access to the maxillary sinus but with a substantial likelihood of intra- and post-operative complications^3^. Access to the challenging areas within the maxillary sinus by traditional endoscopic approaches is very difficult. Possible endoscopic options are the application of a 70° endoscope following a type III sinusotomy, endoscopic standard medial maxillectomy and modified Denker’s and by way of a canine fossa trepanation^4–6^.

Zhou et al.^7^ introduced the endoscopic pre-lacrimal window approach (PLWA) which makes a wide access to the maxillary sinus available by removing the parts of the medial maxillary wall located anterior to the nasolacrimal duct while keeping the lacrimal system and inferior turbinate undamaged. A crucial factor for the performance of a PLWA is the length of the bony window anterior to the nasolacrimal duct. This bony pre-lacrimal window (BPLW) extends from the anterior wall of the maxillary sinus to the anterior border of the nasolacrimal duct and represents the medial wall of the pre-lacrimal recess. The length of the BPLW varies widely among studies^8–12^. Recent studies reported that the BPLW is significantly wider in males than in females—indicating gender-related differences^8,9^. However, the authors stated that further research is certainly warranted to investigate the hypothesis of gender-specific differences, especially as the length of the BPLW shows a great variability.

The amount of surgical effort during a PLWA depends vastly on the width of BPLW. For that reason, Simmen et al.^11^ recently classified 3 feasibility types according to the BPLW length. Type I corresponds to a distance of < 3 mm and renders the PLWA as less feasible due to the requirement of significant bone removal and tear sac dislocation in order to acquire just a limited access to the anterior maxillary sinus wall. A distance of 3–7 mm is defined as Type II and allows a PLWA with higher accessibility but solely with bone work together with lacrimal sac dislocation. Type III (> 7 mm) requires only little bone excision and provides a wide visualization of the most challenging areas within the maxillary sinus. To the best of our knowledge, there is no study in literature which investigated gender-related differences in the prevalence of Simmen’s feasibility types.

The primary aim of this study was to evaluate the antero-posterior length of the BPLW according to gender in an Austrian population. The primary null hypothesis states that there is no difference in BPLW length between females and males. The primary alternate hypothesis is that males exhibit a higher BPLW length in comparison to females. The second aim of this study was to ascertain the prevalence of Simmen’s feasibility types according to gender. The secondary null hypothesis is that males and females show no differences in the feasibility type distribution. The secondary alternate hypothesis suggests that females present a higher prevalence of type I whereas type II and III occur more frequently in males.

## Materials and methods

### Subjects

A retrospective chart review of a consecutive series of paranasal computed tomography (CT) scans performed on Austrian patients, who were admitted between January 2019 and December 2019 to the Department of Otolaryngology, Medical University of Graz, to undergo functional endoscopic sinus surgery (FESS), was performed. Inclusion criteria were a diagnosis of chronic rhinosinusitis sine nasal polyposis (CRSsNP) and an age of at least 18 years. Patients with a medical history of previous FESS and insufficient visualization of the bony landmarks were excluded.

### Computed-tomography imaging

Imaging was performed on high-resolution CT scanners by using axial 1.5-mm cuts and a multiplanar reconstruction technique. A standardized CT protocol that covers all the paranasal sinus from the upper end of the frontal sinus to the maxillary alveolar process was used for imaging. Bone window was selected for examination. Every CT scan was analyzed in the institutional picture archiving and communication system (PACSview) by experienced rhinologists. Provided CT figures in this manuscript were edited by using PaintShop Pro, version 22.0 (Corel, Ottawa, Ontario).

### Measurements

**Figure 1:**
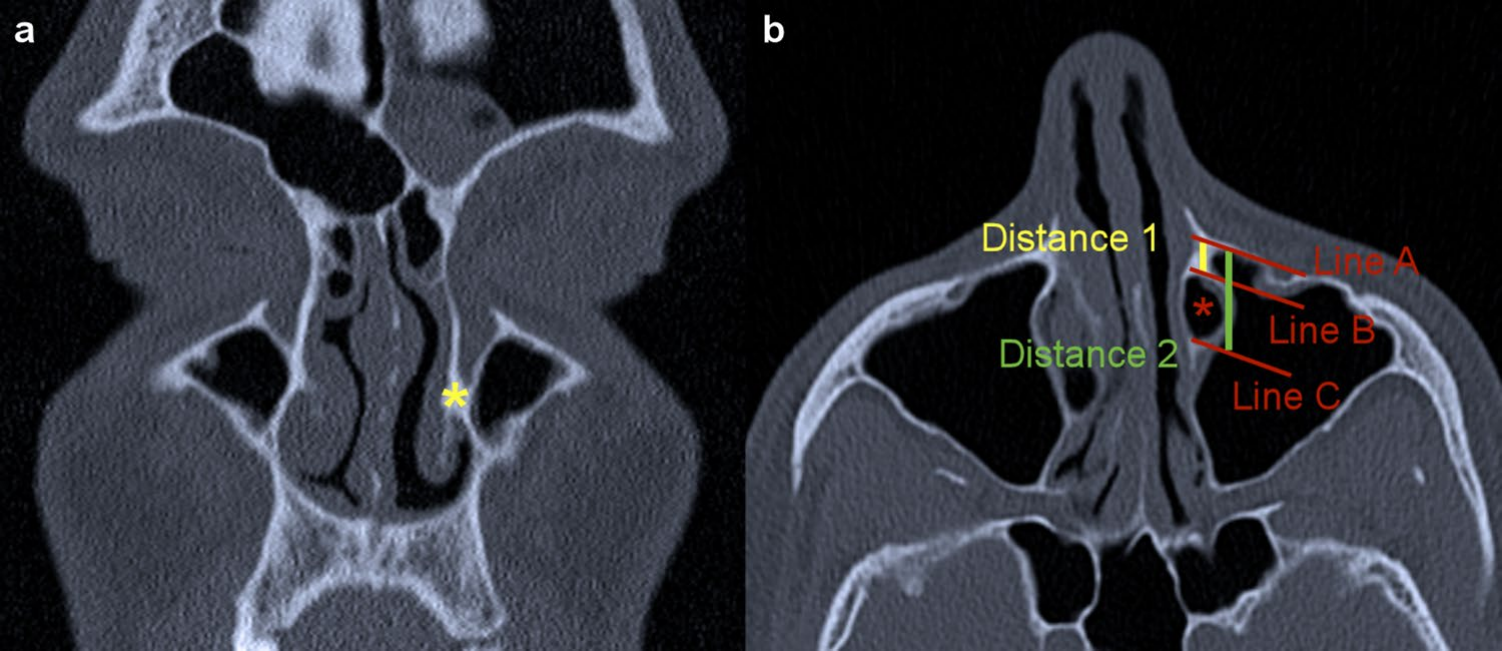
Methodology of measurements. (**a**) The yellow asterisk marks the anterior insertion point of the inferior turbinate into the frontal process of the maxilla, determined in the coronal CT plane. (**b**) The yellow line displays the distance between the anterior maxillary sinus wall and the anterior border of the nasolacrimal duct (Distance 1). The difference between distance 1 and distance 2 (green line) represents the width of the nasolacrimal duct (red asterisk). (**b**) Depicts the corresponding axial CT image to figure a.

**Figure 2:**
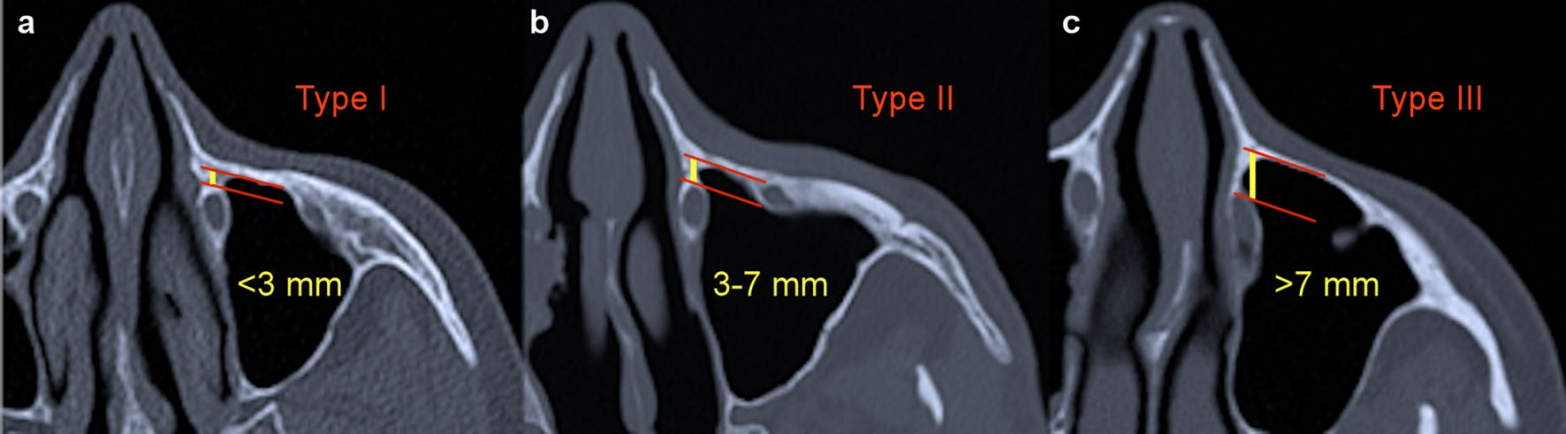
Simmen’s feasibility classification according to the distance between the anterior maxillary sinus wall and the anterior border of the nasolacrimal duct. (**a**) Type I (< 3 mm). (**b**) Type II (3–7 mm). (**c**) Type III (> 7 mm).

Measurements were taken in concordance with Simmen et al.^11^. At first, the point where the inferior turbinate embed into the frontal process of the maxilla was identified in the coronal plane (Fig. 1a). In the corresponding axial plane (Fig. 1b), a tangential line through the anterior wall of the maxillary sinus was then placed (line A). In parallel to this line, two further lines were drawn running through the anterior (line B) and posterior (line C) border of the nasolacrimal duct. The calculated distance between line A and B (distance 1) represented the antero-posterior length of the BPLW. The difference between distance 1 and the distance from the anterior maxillary sinus wall to the posterior border of the nasolacrimal duct (distance 2) represented the width of the nasolacrimal duct. Distance 1 was used to determine the type of PLWA feasibility (Type I: < 3 mm, type II: 3–7 mm, type III: > 7 mm), see Fig. 2.

### Study outcome measures

The primary study outcome was the antero-posterior length of the BPLW. The distribution of the feasibility types according to Simmen et al.^11^ represented the secondary study outcome. Feasibility types were additionally termed as follows: type I = major surgical effort, type II = moderate surgical effort, and type III = minor surgical effort. Both study outcomes were analyzed for the total cohort and separately for males and females.

### Minimum required sample size calculation

Sample size calculation was performed using nQuery statistical software, version 6.0 (Statsols, Cork, Ireland). Due to the fact, that the primary study outcome measure shows a large variability in literature, we evaluated a consecutive pilot series consisting 15 females (n = 30) and 15 males (n = 30) in order to assess the minimum required sample size. All patients in the pilot series met the study eligibility criteria. In this pilot series, females and males presented a BPLW length of 4.2 ± 2.3 (95% CI 3.3–5.1) and 2.9 ± 2.0 (95% CI 2.1–3.6), respectively. Type of power analysis was a-priori: the calculation was highly powered at 95% with a type I error of 5% (α = 0.05). Taking these parameters into account, the minimum required sample size was 72 per gender group for a total of 142. For the secondary study outcome, post-hoc power analysis was used.

The inclusion of both, the right and left side separately, sometimes may cause the doubling number issue. However, even if the face is symmetric, bilateral inconsistency of the paranasal sinuses can be present. A previous trial has reported a bilateral inconsistency of 15% in the size of the BPLW^12^. For that reason, we included each side separately, similar to the previously conducted studies^8–12^.

### Statistical analysis

SPSS statistical software, version 25.0 (IBM, Armonk, NY) was used for statistical analysis. Statistical significance level was set at *p* < 0.05, two-sided. Continuous variables are presented as means ± standard deviations and 95% confidence interval (CI). Categorical variables are expressed as absolute numbers and percentages. Shapiro–Wilk Test was used to evaluate data for normal distribution. As all continuous variables in the present study were normally distributed, unpaired t-test was used to compare continuous parameters between gender groups. Levene test was utilized to check for homogeneity of variances. In presence of variance inhomogeneity, robust Welch’s t-test was used. The effect size of statistically significance differences in unpaired t-tests was expressed by Cohen’s d. For comparison of categorical variables, Chi-squared test was utilized. As the contingency table in the present study consists 3 × 2 variables, Cramer’s V-coefficient was used to assess the effect size of the overall statistically significant difference in Chi-squared analysis. Post-hoc Z-tests in Chi squared analysis were performed with Bonferroni adjustment in order to correct multiplicity (corrected α = 0.05/3 = 0.017). Effect size of Chi squared post-hoc tests was determined with the φ-coefficient.

### Ethical considerations

All patients gave their written informed consent. The study was approved by the local ethics committee of the Medical University of Graz (32-462 ex 19/20) and was performed in accordance with the ethical guidelines of the Declaration of Helsinki.

## Results

A consecutive series of 128 paranasal CT scans of adult patients diagnosed with chronic rhinosinusitis sine nasal polyposis was evaluated for study eligibility. 10 patients were excluded due to a prior FESS in the medical history. Further 18 patients were excluded by reason of insufficient visualization of the medial maxillary sinus wall or nasolacrimal duct. At the end, a total of 50 females (n = 100) and 50 males (n = 100) were included in the study. The mean age of the total cohort was 53 ± 16.3 (95%CI: 50.7–55.2) years. According to gender groups, females and males had a mean age of 51.1 ± 16.2 (95% CI 47.8–54.3) and 54.9 ± 16.2 (95% CI 51.7–58.1) years, respectively. There was no statistically significant difference in mean age between gender groups (M^diff^ = 3.8, 95% CI − 0.6–8.3, *p* = 0.097).

### Total cohort analysis

For the total cohort (n = 200), the mean length of the BPLW (distance 1) was 3.7 ± 2.5 mm (95% CI 3.4–4.1). The average distance from the anterior maxillary sinus wall to the posterior border of the nasolacrimal duct (distance 2) was 11.1 ± 2.3 mm (95% CI 10.7–11.4). The mean width of the nasolacrimal duct was determined to be 7.3 ± 1.6 mm (95% CI 7.1–7.5). Feasibility type I, II and III occurred in 77 (38.5%), 99 (49.5%) and 24 (12%) patients, respectively.

### Gender specific analysis

Detailed results according to gender (n = 100 per group) are depicted in Table 1. Regarding the primary study outcome, males presented a 1.5 mm (95% CI 0.8–2.2) higher BPLW length in comparison to females reaching significance [t(198) = 4.4, *p* < 0.0001, d = 0.622]. The distance from the anterior maxillary sinus wall to the posterior border of the nasolacrimal duct (distance 2) was significantly greater in males than in females [M^diff^ = 1.8, 95% CI 1.2–2.4, t(191.8) = 5.8, *p* < 0.0001, d = 0.828]. There was no statistically significant difference in width of the nasolacrimal duct between gender groups [M^diff^ = 0.2, 95%CI − 0.1–0.7, t(198) = 1.2, *p* = 0.212].

**Table 1:** Patient’s clinical characteristics according to gender groups.

Parameter	Females (n = 100)	Males (n = 100)	*p*-value
BPLW length, mm	3 ± 2.3 (2.5–3.4)	4.5 ± 2.5 (4–5.1)	< 0.0001*
Distance 2, mm	10.2 ± 2 (9.8–10.6)	12 ± 2.4 (11.5–12.5)	< 0.0001*
Width of nasolacrimal duct, mm	7.2 ± 1.8 (6.8–7.5)	7.5 ± 1.5 (7.2–7.8)	0.212
**Simmen‘s feasibility types**			
Major surgical effort (type I)	53%	24%	< 0.0001**
Moderate surgical effort (type II)	39%	60%	0.003**
Minor surgical effort (type III)	8%	16%	0.081

*BPLW* bony pre-lacrimal window. Continuous variables are presented as means ± standard deviations and 95% confidence interval (CI). Categorical variables are expressed as percentages.

*Represents statistically significance at the 0.05 α-level.

**Represents statistically significance at the Bonferroni adjusted significance level (α = 0.05/3 = 0.017).

With regard to the secondary study outcome measure, a statistically significant difference in the distribution of PLWA feasibility types between gender groups was found [χ^2^(2) = 18, *p* < 0.0001, V = 0.300, power = 97%]. Bonferroni-adjusted post-hoc Z-tests revealed that the need of major surgical effort (type I) occurred significantly 29% more frequently in females [χ^2^(1) = 17.7, *p* < 0.0001, φ = 0.300, power = 98%], whereas the requirement of moderate surgical effort (type II) was significantly 21% more prevalent in males [χ^2^(1) = 8.8, *p* = 0.003, φ = 0.210, power = 84%]. The necessity of minor surgical effort (type III) was 8% higher in males compared to females, however, this difference was statistically not significant at the Bonferroni adjusted α-level [χ^2^(1) = 3, *p* = 0.081].

## Discussion

The PLWA provides a wide visualization of the maxillary sinus and allows broad surgically access to the difficult reachable regions within the maxillary sinus with a very low morbidity rate^7^. In a recent study, the PLWA was compared to conventional methods (traditional endoscopic, open and combined) with regard to treatment results of surgically resected inverted papillomas within the maxillary sinus. No recurrence was found in the PLWA group, while conventional methods yielded a 16% recurrence rate^13^. Tran et al.^14^ reported a case series of surgically treated maxillary sinus hemangiomas via PLWA. In all cases, the authors could resect the benign maxillary sinus tumor completely without intra- and post-operative complications. Zhou et al.^15^, the research group who initially introduced the PLWA, reported in a large multicenter study the clinical outcomes of surgically resected inverted papilloma of the maxillary sinus via PLWA over a 10-year time period. A recurrence of the inverted papilloma emerged solely in 7% of the cases. No intraoperative complications or damage to the nasolacrimal duct occurred. Regarding post-operative complications, after 1 year of follow-up 7% of the patients experienced mild paranasal numbness and 5% presented a mild collapse of the ala of the nose. Hildenbrand et al.^16^ investigated the long-term outcome of patients with inverted papillomas of the maxillary sinus treated with PLWA. After a median follow-up period of 3.8 years, no recurrences were found. Besides maxillary sinus pathologies, the PLWA additionally allows endoscopically access to pathologies located in the pterygopalatine fossa and infratemporal fossa^17^.

The PLWA uses the part of the medial maxillary sinus wall, which is located anterior to the nasolacrimal duct, as surgically window to gain access to the maxillary sinus. The antero-posterior width of this bony window is therefore crucial for the performance of a PLWA. This length varies widely among studies. Simmen et al.^11^ reported the first data of the antero-posterior BPLW length. The authors measured in 100 Swiss individuals the distance between the anterior maxillary sinus wall and the anterior border of the nasolacrimal duct at the insertion point of the inferior turbinate attachment to the lateral wall of the nasal cavity. In their study, this distance was 4.24 ± 2.40 mm on average. A recent study from the U.S. by Kashlan and Craig^8^ performed more comprehensively measurements. In an ethnically inhomogeneous population (86 Whites, 40 Blacks and 5 Asians) the antero-posterior BPLW width was determined at 3 different vertical levels. (1) At the most inferior point of the nasolacrimal duct, (2) at the junction of the lacrimal sac and the nasolacrimal duct which represents the superior measurement, and (3) at the halfway level between the inferior and superior measurements. The authors observed a significantly decrease in mean length from inferior to superior (Inferior: 8.4 mm, middle: 7.6 mm and superior: 5.5 mm). The inferior measurement is comparable to the mean BPLW length from the swiss study^11^, which was nearly half the size (8.4 mm vs. 4.24 mm). Interestingly, a comparison between ethnicity groups in the U.S. study revealed no significant differences in BPLW lengths at each level. However, the sample size differed widely between ethnicity groups. Hence, a sufficient conclusion cannot be drawn from this analysis^8^. A Polish research group used the same methodology as Simmen et al.^11^ and determined in their 125 patients a mean BPLW length of 4 mm, which is quite similar to the swiss study^9^. Contrarily, Lock et al.^10^ evaluated the width of the BPLW in 100 Chinese individuals by using Simmen’s methodology and found a mean length of 6.64 ± 2.3 mm—indicating ethnic differences between Orientals and Caucasians. In order to generate comparable results, we adopted the measurements method from Simmen et al.^11^. In the present study, the mean antero-posterior BPLW length in an Austrian population was determined to be 3.7 ± 2.5 mm (95% CI 3.4–4.1), which is quite similar to the results of the other Caucasian studies^9,11^. However, further research with larger sample sizes and a uniform methodology is warranted to investigate the hypothesis of ethnic differences in BPLW length.

Recent studies reported gender-related size differences of the BPLW. Sieskiewicz et al.^9^ found a significantly greater BPLW length in males (4.8 mm) than in females (3.4 mm). Kashlan and Craig^8^ observed as well that the antero-posterior BPLW width, measured at the most inferior point of the nasolacrimal duct, was significantly higher in males compared to females (9.3 mm vs. 7.7 mm). The authors suggested that sexual dimorphism of the piriform aperture may be the reason for gender differences in BPLW size. In our study, females and males had a mean antero-posterior BPLW length of 3 ± 2.3 mm and 4.5 ± 2.5 mm, respectively. We were able to reject our null hypothesis with a 95% power and accept the alternate hypothesis as the determined mean difference of 1.5 mm (95% CI 0.8–2.2) was statistically significant at the 0.05 level (p < 0.0001) with an effect size of d = 0.622. However, statistically significance does not mean clinically significance automatically. In all the above-mentioned studies, including the present one, the mean antero-posterior distance between the anterior maxillary sinus wall and the anterior border of the nasolacrimal duct measured in males, as well as in females, at least 3 mm—a window size enough to conduct a PLWA. Moreover, the mean difference in BPLW length between females and males was not higher than 2 mm. As Kashlan and Craig^8^ also concluded, from our point of view, even if this gender-specific difference in BPLW length was statistically significant, it does not have a huge influence on the decision whether to conduct a PLWA or a conventional approach.

Besides the question whether to perform a PLWA or not, the surgeon must evaluate the feasibility of a PLWA preoperatively. This depends vastly on the distance of the BPLW. In cases of a small BPLW, a significant bone resection and nasolacrimal duct dislocation or even removal with a higher likelihood of morbidity is required to conduct a PLWA. For that reason, Simmen et al.^11^ proposed a feasibility classification for the PLWA in order to determine the percentage of individuals in whom a PLWA can be conducted effortlessly. In this classification, the authors categorized the BPLW into 3 types according to its antero-posterior length: Type I: < 3 mm, type II: 3–7 mm, and type III: > 7 mm. In their study, a type I, who requires a high amount of bone resection and a complete nasolacrimal duct dislocation or even resection, was found in 31.5%. In 56% of the cases, a limited access (type II) with moderate bone removal and necessity of a partial tear sac dislocation was seen. A wide accessibility and visualization of the difficult areas within the maxillary sinus with minor surgical effort (type III) was found in 12.5%. Contrarily, Lock et al.^10^ reported a much higher percentage of PLWA feasibility in a Chinese population. The authors found a feasibility rate of 6.5% for type I, 53.5% for type II and 39.5% for type III. Our results of the feasibility types were quite similar compared to Simmen et al.^11^. We observed a type I in 38.5% while a type II and III occurred in 49.5% and 12%, respectively. None of these studies analyzed their results in terms of gender. We therefore present the first data of PLWA feasibility regarding gender distribution. The distribution of feasibility types between females and males differed statistically significant at the 0.05 level (p < 0.0001) with an effect size of V = 0.300. According to post-hoc power analysis, we were able to reject the null hypothesis with a 97% power and accept the alternate hypothesis. The requirement of a high amount of bone work and a complete nasolacrimal duct dislocation (type I) in order to gain only limited access to the maxillary sinus was 29% higher in females (*p* < 0.0001, φ = 0.298). This statistically significant difference was highly powered at 98%. In contrast, the need of moderate bone resection and partial nasolacrimal duct dislocation (type II) was 21% higher in males (*p* = 0.003, φ = 0.210, power = 84%). The necessity of only minor surgical effort to achieve a wide visualization of the maxillary sinus (type III) was twice as high in males compared to females (8% vs. 16%, *p* = 0.081). Even if the latter difference was statistically not significant, from our perspective, it is of clinically importance. In conclusion, our data indicates that females require more significant surgically effort during a PLWA in order to gain access to the maxillary sinus. These results are highly informative as a high amount of bone removal and nasolacrimal duct dislocation are associated with an increased probability of intra- and post-operative complications.

Nevertheless, there are some limitations that should be addressed. First, the present study was retrospectively in nature. Second, even though our sample size was large enough in order to obtain highly powered significant results according to our power analysis, we are aware that our total study size of 200 maxillary sinus may cause selection bias when extrapolating to the population.

## Conclusions

The present study revealed gender-specific differences in feasibility of a PLWA. In order to conduct a PLWA, the necessity of major bone resection and complete nasolacrimal duct dislocation was 29% higher in females whereas males had a 21% greater prevalence in the requirement of moderate bone removal. The need of minor bone work to gain wide access to the maxillary sinus was twice as high in males compared to females. These results suggest that females require more significant surgical effort during a PLWA which results in a higher likelihood of intra- and post-operative complications. Further prospective trials with larger sample sizes are warranted to confirm the present study findings.

## Data Availability

The datasets generated and analyzed during the current study are available from the corresponding author on reasonable request.
